# In this issue: Volume 114, Issue 5, May 2023

**DOI:** 10.1111/cas.15819

**Published:** 2023-05-02

## Frontiers in mass spectrometry–based clinical proteomics for cancer diagnosis and treatment

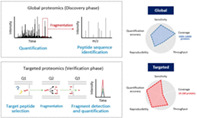

The detection of cancer biomarkers, primarily proteins, plays a significant role in the treatment of cancer. Proteins are responsible for most of our body's biochemical functions. To understand how their expression and functions are altered in cancer, their accurate measurement in clinical samples becomes critical. However, the expression levels of different proteins in clinical samples can vary significantly and are therefore difficult to analyze.

Mass spectrometry (MS) is a promising technology used to measure changes accurately in the levels of proteins present in body fluids, cells, and tissues. In samples from cancer patients, these include various cancer‐related proteins, their cancer‐specific forms, as well as proteins whose levels fluctuate as the cancer progresses.

In this article, Yoshimi Haga et al. from Japan reviewed recent advances in MS technology and their impact on cancer research and treatment.

One such advancement in MS technology is the prostate‐specific antigen glycoform analysis for improving diagnostic accuracy in patients with prostate cancer. With improved analytical techniques and more efficient sample analysis, glycoproteomics is expected to become a promising tool for biomarker discovery.

More recently, the detection of mutations in cancer cell proteins has flourished with improvements in MS sensitivity. At present, MS is the only analytical technique that can identify the amino acid sequences of tumor‐specific small fragments of proteins known as neoantigens among immunopeptides, which help in the immune recognition of cancer cells as non‐self, i.e., abnormal cells.

Additionally, more sensitive and innovative technologies are emerging in the field of proteomics. These are expected to identify and characterize rare cells, such as cancer stem cells, while detecting the early stages of drug resistance.

In summary, advances in MS‐based proteomic profiling are expected to provide clinically helpful information for understanding the mechanisms of cancer progression, cancer diagnosis, and drug development. They are also expected to make a significant contribution in the area of personalized medicine.

https://onlinelibrary.wiley.com/doi/full/10.1111/cas.15731

## HNRNPC suppresses tumor immune microenvironment by activating Treg cells promoting the progression of prostate cancer

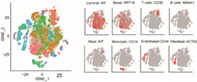

Prostate cancer is prevalent among men and is characterized by the development of malignant cells in the prostate gland, which is a part of the male reproductive system. Surgical removal of the malignant mass followed by chemotherapy can treat manifestations of the disease that remain localized. However, in most cases, cancer metastasizes through the body into other organs, and leads to the development of a fatal, castration‐resistant form of cancer.

While immunotherapy is gaining prominence as a promising therapeutic approach for prostate cancer, not all patients respond equally to the treatment. Scientists have attributed the variable success rates of the treatment to the unique immune tumor microenvironment (TME) in each patient. Thus, exploring the regulatory mechanisms of the TME could be key to perfecting and customizing immunotherapy.

In this paper, Cheng et al. focused on one such mechanism – the methylation of N6‐methyl adenosine (m6A) – a biochemical process that plays a prominent role in the progression of multiple cancers. The researchers set out to understand how m6A and the immune system influence each other in regard to prostate cancer progression and find biomarkers that would enable an improved treatment response.

They analyzed the regulatory factors of m6A and how they affected the immune cells present in the TME. Based on the m6A gene profiles obtained from The Cancer Genome Atlas database and simulations, they were able to identify the hub genes and pathways crucial to the immune response against prostate cancer. Validating their findings through in vitro experiments and bioinformatics analyses, the researchers discovered that the HNRNPC protein encoded by *HNRNPC*, which was identified as the hub gene, played a key role in inhibiting the immune response to cancer cells.

They also found that *HNRNPC* interacts with m6A to promote the genetic modification of certain RNA domains and activates the infiltration of regulatory T cells, a type of immune cell, in the TME.

In summary, the study identifies *HNRNPC* as a genetic biomarker of prostate cancer, suggesting that it could be targeted to activate the immune response against the disease. This insight, in turn, could potentially advance immunotherapy strategies for managing prostate cancer.

https://onlinelibrary.wiley.com/doi/full/10.1111/cas.15745

## Crizotinib‐based proteolysis targeting chimera suppresses gastric cancer by promoting MET degradation

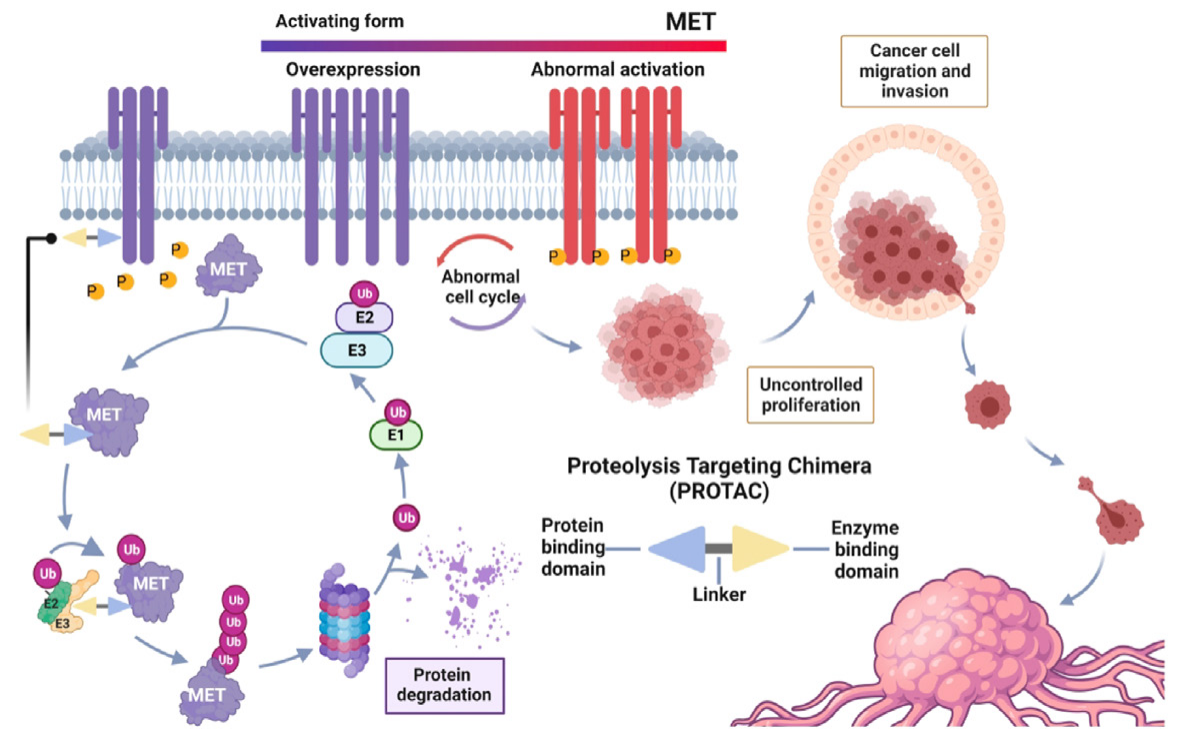

Stomach cancer, one of the most common cancers affecting people worldwide, poses a significant challenge because of its late‐stage diagnosis and poor survival outcomes. A common finding among late‐stage stomach cancer patients is the overexpression of receptor tyrosine kinase MET. While certain drugs, like crizotinib, can bind and inhibit MET, they have not been approved for treatment because the protein can acquire resistance to these.

An emerging therapeutic strategy called proteolysis targeting chimera (PROTAC) offers hope for the development of MET‐targeted therapies. PROTAC refers to small molecules that consist of two linker‐separated components—a targeting molecule that seeks out a specific protein and a second molecule that triggers the destruction of that protein. When the PROTAC molecule locates the target protein, it recruits a special protein called E3 ligase, which is part of the protein breakdown machinery of the cell, causing the degradation of the target protein.

To assess the viability of this strategy in controlling MET levels, researchers successfully designed a series of PROTACs that incorporated the MET‐specific drug crizotinib as the targeting molecule and evaluated their efficacy against stomach cancer cell lines. Findings reveal that the most efficient PROTAC (PRO‐6E) successfully eliminated MET proteins and inhibited the growth and spread of MET‐overexpressing stomach cancer cell lines. Additionally, PRO‐6E retained its anti‐cancer activity and was well‐tolerated when orally administered in mouse models with human stomach cancer cells having high MET levels. These findings highlight the potential of PRO‐6E as the first oral PROTAC in treating stomach cancers characterized by MET overexpression.

In conclusion, the use of crizotinib‐based PROTACs represent a promising new approach for the treatment of MET‐positive stomach cancer. This therapy has the potential to improve patient outcomes and offers a personalized treatment approach for individuals suffering from this condition. While further research is needed to fully understand the long‐term efficacy and safety of this treatment in humans, the results so far prove highly promising in transforming the treatment landscape for patients with stomach cancer.

https://onlinelibrary.wiley.com/doi/full/10.1111/cas.15733

